# The Power of Social Attribution: Perspectives on the Healing Efficacy of Ayahuasca

**DOI:** 10.3389/fpsyg.2021.748131

**Published:** 2021-10-28

**Authors:** Bernd Brabec de Mori

**Affiliations:** ^1^Institute of Musicology, University of Innsbruck, Innsbruck, Austria; ^2^Institute for Social and Cultural Anthropology, University of Marburg, Marburg, Germany

**Keywords:** ayahuasca, constructivism, indigenous knowledge, stereotypes, colonialism, appropriation

## Abstract

During the last decades, ayahuasca gained much popularity among non-Indigenous and out-of-Amazonia based populations. In popular culture, it has been advertised as a natural remedy that was discovered by Indigenous peoples ante millennia and that has been used for shamanic healing of all kinds of ailments. This “neo-shamanic,” and often recreational, use of ayahuasca, however, has to be distinguished from traditional Indigenous praxes on the one hand, and, on the other hand, from medical investigation in the modern world. The former, Indigenous use mainly understands ayahuasca as an amplifying power for interacting with non-human beings in the animal, plant, or spirit realms. Within this paradigm, efficacy is not dependent on the drug, but on the correct communication between the healer (or sorcerer) and the non-human powers that are considered real and powerful also without resorting to ayahuasca. The latter, modern mode of understanding, contrastingly treats the neurochemical processes of MAO inhibition and dimethyltryptamine activity as trigger mechanisms for a series of psychological as well as somatic responses, including positive outcomes in the treatment of various mental conditions. I argue that there is an ontological incommensurability occurring especially between the Indigenous and medicinal concepts of ayahuasca use (with recreational use in its widest understanding trying to make sense from both sides). Modern medical applications of ayahuasca are so fundamentally different from Indigenous concepts that the latter cannot be used to legitimate or confirm the former (and vice versa). Finally, the deep coloniality in the process of appropriation of the Indigenous by the modern has to be questioned and resolved in any case of ayahuasca application.

## Introduction

In recent times, ayahuasca has been in use by at least three large and overlapping groups: traditional Indigenous and Mestizo people within their communities, neo-shamanic and recreational users all over the world, and patients of mental health facilities in the realm of modern medicine and psychiatry. In this perspective paper, I will show that although all these forms of use are legitimate and meaningful, they are still situated in a deep colonial structure of power relations and appropriation. For example, in neo-shamanic and medical use, traditional healers are often employed, who are then missed in their original communities. In addition, it seems that the efficacy of ayahuasca in both neo-shamanic and medical use draws primarily from ascriptions of alterity to the substance: why do modern users so often resort to constructed images of Indigeneity, naturalness, and ritual when drinking or administering ayahuasca, while at the same time its efficacy is ascribed to its pharmacology, or to “doing ayahuasca”? In order to answer this question, I will compare traditional Indigenous concepts with neo-shamanic or recreational, and modern medical concepts of attributing efficacy to this substance.

From the first half of the 20th Century on, reports of Westerners concerning ayahuasca placed it as a “medicine” within the construction of “ayahuasca shamanism,” that is a health-related use of this substance, although Indigenous or Mestizo people then mainly used ayahuasca for divination, (counter-)witchcraft, warfare, and communal religious rituals (Gow, 1994; Bianchi, 2005; Brabec de Mori, 2011). The idea that ayahuasca was used for “curing,” although not yet literally for medical purposes, probably dates back to the rubber boom and its exploitation and genocidal mistreatment of Indigenous populations that had to be “healed” in a political-metaphysical sense of empowerment and reconciliation (Taussig, 1987; Byrne, 2017). Indigenous medical concepts are much older and deeply rooted in an animist worldview and complex techniques of contacting, socializing with, and dealing out reciprocities with non-human spirits, animals, or other entities; techniques that completely lack any necessity of ayahuasca use.

Despite the constructed quality of “ayahuasca shamanism,” it seems impossible to shed off implicit assumptions in contemporary Western or “modern” applications about ayahuasca’s “mythical,” “spiritual,” “shamanic,” “ritual,” “Indigenous,” “entheogenic,” “ecological,” and similar qualities that are ascribed to this complex (see Gearin, 2015; Fotiou, 2020a,b). Especially neo-animist renderings of “mother ayahuasca” or “teacher” and “master plants” are impressively popular among Westerners. I argue, therefore, that ayahuasca’s distinctive successes in both popular culture and medical investigation are indebted mainly to said social attributions of healing power grounded in intrinsic assumptions of Alterity.

I use the term ‘‘social attribution’’ to denote the *discourse about* a specific item, in this case, ayahuasca, and what it is assumed to do to its users.^1^ Different forms of discourse shape differing opinions about the item and thus inform different certainties about its qualities. In all cases discussed here, ayahuasca as a substance is constituted by basically the same, or similar, human pharmacology, but the qualities that are attributed to this substance show significant variation.

In the following, I will describe three modes in which ayahuasca is used. Quite naturally, when one constructs categories, exceptions from the rule will be found: there are many instances where the three modes overlap and interact, and in addition, though very rarely, some people may use ayahuasca in other modalities than described here.

## Social Attributions in Indigenous Traditional Use

It is difficult to present Indigenous concepts around ayahuasca in a nutshell, so I will exemplarily explain traditional ayahuasca use among the Peruvian Shipibo-Konibo. This is where I conducted systematic fieldwork from 2000 to 2006, continued through visits until 2019. I describe the healing-sorcery practices as observed among Shipibo-Konibo healers (*médicos*) who in the early 2000s worked among their communities, but not (yet) with tourists or visitors.

In traditional Shipibo medicine, an apprentice would embark on lengthy “diets” (*samá*; cf. Illius, 1987; LeClerc, 2003; O’Shaughnessy and Berlowitz, 2021) that do not involve the intake of ayahuasca but of other plants, and seldom animal or inorganic substances. These are ingested before and during a span of time when the apprentice would retire from much of social contacts and follow a set of alimentary and social taboos. Note here, that ‘‘diets’’ are also prescribed for patients after a curing session (in order not to disturb the songs that are thought to linger in the body), or for healing processes that apply any plant preparations. ‘‘Diets’’ are likewise essential for many forms of learning, e.g., for becoming a good hunter, for producing precious artwork, for toddlers to walk and talk more quickly, for being a good soccer player or musician, and so on. During a ‘‘diet’’ devoted to becoming a healer-sorcerer, however, the apprentice should make contact with the humanoid entities of the plant ingested, who are called ‘‘owners’’ (*ibo*) or ‘‘spirits’’ (*yoshin*), in dreams or wake-state visions. From these, the disciple obtains their powers which often take the forms of songs. Between ‘‘diets,’’ the apprentice would accompany his^2^ teacher in curing sessions and thereby practice working with him and learn to apply what he obtained from his “diet.” There was no formal initiation, so at some point the apprentice would start conducting healing or sorcery sessions on his own, thus stepping into the competitive ring of healer-sorcerers. Illness was understood as a reciprocal process, stemming from a source, which, in order to heal, has to be tricked, seduced, or overthrown. In cases when a competing specialist would be held to be the original causer of an illness, from the others’ perspective, “my healer” is “his sorcerer” (Brabec de Mori, 2017).

Efficacy is attributed to the power of a healer from close kin who accomplished many lengthy “diets,” has vast social relations with animals, plants, and spirits, and knows a great repertoire of magical songs – the main means of interacting with his non-human allies, and their foes. The correct use of melodies, language, codes, and metaphors was crucial. The powerful voice (see Brabec de Mori, in print) would be obtained through “diets,” too. I repeat that the use of ayahuasca was totally optional, restricted to the specialist himself, and the most powerful healer-sorcerers, the *meraya*, did not use it at all.

It is important to note that in order to accomplish “diets,” and to be instructed by a teacher, one had to “stand firmly on both legs,” as one specialist put it. Therefore, the intake of ayahuasca was (ideally) socially restricted to healthy, psychologically and socially established well-trained individuals, because, as indicated above, only they would drink the brew, while patients or laypeople would never touch it – “why should I drink this, I am not a healer!”

Among all the Indigenous healers I worked with, high-intensity hallucinations were avoided. Most healers would drink fairly low doses in order to only “open up the world” (*nete kepenti*). They would retain control of their condition in order to channel their songs’ power toward the patient. Finally, vomiting was not a topic. During around 100 ayahuasca sessions I witnessed among traditional Shipibo healers, no single one would ever vomit. Ayahuasca was considered a delicate tool to more easily reach the spirit world, nothing more, nothing less.

## Social Attributions in Neo-Shamanic and Recreational Use

In Indigenous use prior to the ayahuasca boom, the healer would take the substance, but not the sick. This constitutes the most prominently marked difference between Indigenous and modern understandings of ayahuasca: ayahuasca had to – by itself, as a substance – show therapeutic effect, and clients would be those who had to go through the hallucinatory experience. Thus, a “psychologization” of the whole process took place (Brabec de Mori, 2013; Labate, 2014). Remarkably, Indigenous thought attributes efficacy to the healer’s power, knowledge, and experience, while Westerners cannot but attribute efficacy to the substance-as-ingested.

The appearance of Western researchers and later drug and healing tourists and visitors triggered a transformation in Amazonian Indigenous and Mestizo ‘‘traditions’’ toward communal ayahuasca-drinking sessions that became known as ‘‘ceremonies.’’^3^ This newly invented style of ayahuasca use was then exported to North America and Europe, and practically all over the world. Nowadays, a multitude of neo-shamans who either learned from the first generation of out-of-the-Amazon *ayahuasqueros*, or who created their own eclectic ayahuasca ceremony styles, offer their services to a growing general public. Most of these sessions are held exclusively for “white” or other non-Indigenous, non-local people. There is much work about these forms of ayahuasca use in the Amazon and beyond (see Gearin, 2015; Labate et al., 2016; Fotiou, 2020b). I will restrict my analysis here to my fieldwork among White neo-shamans in Shipibo territory, and to phenomena that I consider most distinctive.

In these neo-shamanic contexts, the importance of “diets” is varied, in some cases they are completely omitted (most often in foreign contexts), or they are recommended because the brew would cause stronger hallucinogenic effects after fasting. In the latter cases, the diet has to be held before the ayahuasca session, while in Indigenous use, it should follow the treatment. For “becoming a shaman,” the diet is usually prescribed very much in the Indigenous sense, but most often “mixed” with ayahuasca ingestion, or thought to “strengthen” one’s control of ayahuasca hallucinations. Diets are rarely considered important for anything beyond the ayahuasca complex.

Another main distinction is a certain “white-washing” of the practice: “shamans” are considered “good,” and ayahuasca use is by definition beneficial. Sorcery is not considered inherent (see Fotiou, 2010), and not connected to emotion control or psychological stability. Quite on the contrary, as I observed among “shaman apprentices” in Ucayali, many of them have a history of minor mental disorder or at least some issues with mental or social instability, not by any means “standing firmly on both legs.” Many are seekers, with “alternative” world-views, lacking or avoiding well-defined integrated professional or family life (of course there are some exceptions here). Therefore, ayahuasca by itself and its use are considered to be beneficial to health, spiritual growth, inspiration, and general well-being, promising a possible solution for preconditions like mental problems or social instability.

This results in a very different kind of people drinking ayahuasca compared to Indigenous traditions: mainly people with a background of experience-seeking, of spiritual, psychological, ecological, or socio-economic discontent, or with diagnosed health problems tend to attend such ceremonies (Wolff and Passie, 2018). The attribution of efficacy goes to the substance itself, which is often worshiped as “mother,” “teacher,” or “master,” and to a complex of alleged authenticity in terms of eco-harmony, purity, shamanism, spirituality, and indigeneity, say: colonial style exoticism. Local ayahuasca ceremonies I observed in Central Europe are highly ritualized and exoticized, and immense healing power is attributed to the substance and its immediate effects: heavy vomiting, physical suffering, and harrowing, dreadful visions are seen as necessary for a cathartic experience which in turn is considered foundational for achieving spiritual, mental, or physical well-being.

I could also observe that many White shaman apprentices in Peru were surprisingly uninterested in the lived world of the “non-ayahuasca-drinking” locals, who still constitute the vast majority of Indigenous or Mestizo populations, and their situation of marginalization and often extreme and illness-causing poverty. One shaman apprentice even believed that “they are happy living in that way.” There are many people who do care, of course, but I was harshly surprised that this attitude occurred among all (randomly selected) White shaman apprentices I interviewed in hostels and “albergues” during my fieldwork in 2019.

## Social Attributions in Western Therapeutic and Clinical Use

There is strong and growing support for a general efficacy of so-called “psychedelic,” hallucinogenic drugs including LSD, MDMA, mescalin, psilocybin, ketamine, and others, in the context of treating a variety of non-psychotic mental conditions including, for example, recurrent depression, anxiety and obsessive-compulsive disorders, and drug addiction. Ayahuasca, too, seems beneficial in therapies of various mental disorders (most solid in addiction treatments, see Bouso and Riba, 2014; Palhano-Fontes et al., 2019).

Pioneer studies on the chemistry and human pharmacology of the substance were mostly undertaken in un-controlled settings, they had to deal with the difficult accessibility of the material (Rivier and Lindgren, 1972), or create alternatives to circumvent the problems of remoteness and prohibition (Ott, 1994). Similar problems appeared for investigations about the efficacy of ayahuasca on health-related conditions, though they still have to be acknowledged as most seminal in the field (Grob et al., 1996; Riba et al., 2001). Until today, many studies about the effects of ayahuasca on human health have to be conducted “in the wild” in (more or less legal) ayahuasca ceremonies. Uthaug et al. (2021), for example, “visited 6 ayahuasca retreats, hosted by a single organization, all taking place at several locations in Europe….” The same authors clearly state that “Only ‘students of the ayahuasca school’ (linked to the host organization) were invited to participate in the present single-blind, placebo-controlled study.” That means that the population under study were exclusively European ayahuasca users who had previously decided to devote themselves to “studying” with the “host organization,” which – though un-attended by the authors – carries a strict dogma of ayahuasca’s properties, and students probably feel obliged to reproduce these. Similar limitations (acknowledged by the authors) apply to the “global ayahuasca project” (Sarris et al., 2021) that based its analysis on 11.912 online survey responses by people from all over the world who, obviously, were mostly involved with groups of regular, and biased, ayahuasca users.

Although most clinical studies are methodologically well designed and solid, there is a strong bias to be observed when reviewing the literature, that either researchers, the studied population, or both underlie the abovementioned assumptions that ayahuasca *per se* was a medicine. For example, in Labate and Cavnar’s (2014) influential book *The Therapeutic Use of Ayahuasca*, where some of the finest scientists working on the human pharmacology of the substance contributed chapters, the foreword reproduces ethnographic myths, and rather naïvely states, without any reference, that among Indigenous people “ayahuasca is considered a medicine: the great medicine. Practically, in today’s world, the shamanic model incorporates easily-transferable features, including group structure, attention to set and setting, import of intention, and proper preparation for and integration of the experience” (Grob, 2014: x–xi). In my perspective as a critical ethnographer, this attitude is way colonial. It does not listen to but models a Western imagination of, Indigenous people’s opinions. Most chapters in Labate and Cavnar’s book, and many studies on ayahuasca as a therapeutic tool, cling to un-referenced and never tested assumptions that this substance would have been used by Indigenous people during millennia for the purpose of (spiritual) healing – although this “healing” was recently constructed during the second half of the 20th Century.

A psychedelic experience may, on the contrary, also be disturbing or frightening and even cause lasting mental health problems. This seems to occur very rarely and mostly through triggering pre-existing latent disorders, and could be statistically counterbalanced by positive effects in others (Krebs and Johansen, 2013). Heise and Brooks (2017) report acute intoxication crises after ayahuasca ingestion that were brought to US poison control, including three fatalities. Dos Santos et al. (2017) registered diagnosed psychotic episodes from published literature. Gearin and Calavia Sáez (2021: 147) found that personality disorders, especially “narcissism and related problems of the self” may be exacerbated by ayahuasca use. Although cases of less severe adverse effects may have escaped these studies, an overall beneficiary effect seems evident as such.

In stark contrast to the Indigenous concept, the drug is now ingested by the patients, that is, exclusively by people with diagnosed mental health conditions. The (hopefully) psychologically and socially stable therapist, on the contrary, does not drink the brew together with the client. In most countries, healthy individuals are not allowed to drink ayahuasca while ill people may obtain a legal exception.

In sum, I do not doubt that ayahuasca, along with other hallucinogens despite some counterindications may be very useful in treating certain mental disorders. However, the power of social attributions remains un-attended in many studies (but see e.g., Talin and Sanabria, 2017), and expectations abound – even among the researchers themselves who are often quite fond of their own visionary experiences obtained through the brew, possible leading to a sense of mission, which is rather unsettling in terms of good scientific practice (cf. Grob, 2014: xiii).

## Outlook

People form general beliefs fed by their social environment, in this case, people (including scholars) believe that ayahuasca has intrinsic natural, medical, spiritual, ecological powers that had been suppressed through the Western world’s (often radical) secular, disenchanted life-world history. People with such a general belief are then seeking and creating situations that trigger certain experiences, which in turn enable them to form a personal (or scientific) account, including mental states and personal development, that strengthens the general belief (Van Leeuwen and Van Elk, 2018).

This should not be misunderstood as a generalizing critique on the use and study of ayahuasca and other hallucinogens for therapeutic applications. I do suggest, however, that there are more contextual (e.g., ritualization) and psychological (e.g., beliefs) factors to be understood than hitherto recognized. Furthermore, I think that the three modes of ayahuasca use I briefly presented here are ontologically different. Social attributions of efficacy range from interaction with spirits (requiring an animist ontology, see Descola, 2013), to worshiping neo-indigeneity and ecology (requiring a colonial world and ecological doom), to a substance that is therapeutically effective (a naturalist ontology). Therefore, it is not enough to heed some “cultural” factors. For serious medical studies, research designs have to be separated from Indigenous concepts, and, if possible, from ideologically biased recreational (in the widest sense) applications of ayahuasca. This is difficult indeed, given that many authors are themselves involved in some form of ayahuasca ritual circles, churches, or freelance use.

Finally, the implicit coloniality in virtually all forms of ayahuasca use should largely be recognized, and strategies have to be developed to find a way of fair use of ayahuasca in an anti-colonial sense. Those regions where ayahuasca originated and where most ayahuasca centers are located are among those hardest hit by COVID-19 death tolls. How can this be?

## Data Availability Statement

The original contributions presented in the study are included in the article/supplementary material, further inquiries can be directed to the corresponding author.

## Author Contributions

BB was the sole author and responsible for all the content and manuscript redaction.

## Conflict of Interest

The author declares that the research was conducted in the absence of any commercial or financial relationships that could be construed as a potential conflict of interest.

## Publisher’s Note

All claims expressed in this article are solely those of the authors and do not necessarily represent those of their affiliated organizations, or those of the publisher, the editors and the reviewers. Any product that may be evaluated in this article, or claim that may be made by its manufacturer, is not guaranteed or endorsed by the publisher.

## Footnotes

1 Attribution theory was used in social psychology to understand “folk psychological” judgments of one person about another but has been mostly substituted by discourse analysis (Potter and Edwards, 1990). Attribution theory is nowadays used in organizational sciences for example to describe how laypeople perceive certain brands (Al-Sharif, 2020).

2 I use the male form here, because in traditional Shipibo ayahuasca use, only men would embark on these duties. Female participation was extremely rare but has rapidly been growing since around 2000.

3 The term “ceremonies” recently appeared in the 1990s and has been exclusively used for sessions involving visitors; the same happened with the term “shaman” (for a critical discussion, see Martínez González, 2009).
